# Quantitative Characterization of Microfiltration Membrane Fouling Using Optical Coherence Tomography with Optimized Image Analysis

**DOI:** 10.3390/membranes16020050

**Published:** 2026-01-26

**Authors:** Song Lee, Hyongrak Cho, Yongjun Choi, Juyoung Andrea Lee, Sangho Lee

**Affiliations:** School of Civil and Environmental Engineering, Kookmin University, 77, Jeongneung-ro, Seongbuk-gu, Seoul 02707, Republic of Korea; sophia3374@kookmin.ac.kr (S.L.); rhino@kookmin.ac.kr (H.C.); choiyj1041@gmail.com (Y.C.); jyl20101530@gmail.com (J.A.L.)

**Keywords:** membrane, microfiltration, fouling, optical coherence tomography, image analysis

## Abstract

Membrane fouling reduces permeate flux and treatment efficiency, yet most diagnostic methods are destructive and require offline analysis. Optical coherence tomography (OCT) enables in situ, real-time visualization; however, quantitative image extraction of thin foulant layers is often limited by manual processing and subjective thresholding. Here, we develop a reproducible OCT image-analysis workflow that combines band-pass filtering, Gaussian smoothing, and unsharp masking with a dual-threshold subtraction strategy for automated fouling-layer segmentation. Seventeen global thresholding algorithms in ImageJ (289 threshold pairs) were benchmarked against SEM-measured cake thickness, identifying Triangle–Moments as the most robust combination. For humic-acid fouling, the OCT-derived endpoint thickness (14.23 ± 1.18 µm) closely agreed with SEM (15.29 ± 1.54 µm). The method was then applied to other microfiltration foulants, including kaolin and sodium alginate, to quantify thickness evolution alongside flux decline. OCT with the optimized image analysis captured rapid early deposition and revealed periods where flux loss continued despite minimal additional thickness growth, consistent with changes in layer permeability and compaction. The proposed framework advances OCT from qualitative visualization to quantitative, real-time fouling diagnostics and supports mechanistic interpretation and improved operational control of membrane systems.

## 1. Introduction

Membrane technology has become a well-established and rapidly growing option for various separation processes, particularly in water applications, over the past decades [1]. Its adoption spans various industries, including water treatment, wastewater reclamation, desalination, and produced water treatment [2,3,4]. It is now recognized as a suitable method with which to solve global problems of water scarcity and pollution; its use continues to grow as the demand for water rises [5,6]. Membrane systems offer several advantages over conventional water treatment technologies, including high separation efficiency, compact and modular design, minimal space demand, high process flexibility, high energy efficiency, and easy scalability [7,8,9]. In recent years, the development of advanced membrane materials, including aquaporin-based membranes, carbon-nanotube or graphene-derived membranes, and metal–organic frameworks (MOFs), has opened new possibilities for next-generation membranes, offering enhanced permeability and selectivity [10,11,12].

Nonetheless, membrane technology faces challenges associated with membrane fouling and subsequent performance deterioration [13]. Membrane fouling occurs due to the accumulation of various substances on the membrane surface or within its pores, resulting in reduced permeate flux, compromised product quality, shortened membrane lifespan, and increased maintenance requirements [14,15]. It is exacerbated by complex physical and chemical interactions between feed constituents and the membrane material, leading to surface and pore blockage. The deposited material can be dissolved particles, organic or inorganic molecules, or biological micro-organisms [16]. Since fouling has a significant impact on treated water production and water quality in membrane processes, various research has been conducted to control fouling, including pretreatment, operating condition adjustments, membrane cleaning, and the fabrication of fouling-resistant membranes [14,17,18].

The effective implementation of fouling-control strategies depends crucially on reliable monitoring, which may provide timely, evidence-based insights into the formation, evolution, and impact of deposits on membrane performance [19]. High-resolution monitoring enables early detection of fouling onset well before significant flux decline or pressure increase [20]. By quantifying fouling tendencies in real or near-real time, it is possible to optimize fouling control methods to enhance process stability, minimize downtime, and extend membrane lifespan [21,22]. Moreover, accurate fouling monitoring reduces energy and chemical consumption [23].

There are two categories of fouling-monitoring approaches: invasive (intrusive) and non-invasive (non-intrusive) techniques. Invasive monitoring methods require the interruption of system operations and often involve disassembling the membrane module to directly analyze the membrane and fouling layers [22]. These methods are typically used offline and are rarely applied in practice due to their disruptive nature and potential to damage membrane components [24]. As invasive techniques, direct visualization and scanning electron microscopy (SEM) are commonly used to evaluate the surface morphology and structure of fouling layers, providing detailed information on the chemical makeup and extent of fouling [25]. Nevertheless, invasive methods are expensive, can introduce artifacts, and are not suitable for real-time or continuous monitoring [26,27].

Non-invasive methods allow for in situ, real-time monitoring of membrane fouling without interrupting system operations or damaging the membrane [28]. These techniques have the potential to be used in practice and are preferred for their ability to provide continuous data under realistic operating conditions [29]. As non-invasive methods, direct observation through the membrane (DOTM) [30], including ultrasonic time-domain reflectometry (UTDR) [31], nuclear magnetic resonance (NMR) [32], electrical impedance spectroscopy (EIS) [28], and optical coherence tomography (OCT), have been attempted in previous works. Each method has specific limitations that affect its applicability, resolution, and practicality in real-time and industrial settings. DOTM requires optical access; therefore, it cannot be used in turbid or opaque systems [33]. UTDR requires a significant difference in acoustic impedance between the membrane and fouling layer, which is not always present, especially for organic fouling and polymeric membranes [19]. NMR has limited digital resolution, leading to large errors in estimating fouling-layer thickness [34]. EIS requires electrodes within the membrane cell, which can be invasive and may risk damaging the membrane if not properly managed [35]. OCT may require transparent windows or feeds, restricting its use in turbid or opaque systems [36]. Moreover, most non-invasive techniques are preferably applicable to flat-sheet membrane modules. Even with these limitations, there is a growing interest in non-invasive techniques due to the potential for further advances [29].

Among these, OCT is an emerging imaging technology used to monitor and quantify fouling during the membrane process [37]. OCT acquires cross-sectional structural information by measuring the echo time delay and intensity of backscattered near-infrared light [16,38]. It generates depth-resolved profiles with micrometer-scale axial resolution, capturing the spatial distribution, thickness, morphology, and temporal evolution of fouling layers without disrupting membrane operation [39,40]. OCT can be used as a powerful diagnostic tool to monitor membrane fouling, providing a better understanding of the mechanisms involved, enabling more targeted operation and facilitating the development of predictive control strategies for membrane processes [41,42]. OCT has been applied to monitor fouling caused by various components, including particles [40,43,44], organic matters [45,46], oil emulsion [33], and biofilm [47]. Nevertheless, previous applications of OCT have been limited to flat-sheet membranes, which are not commonly used in industrial scales. While the direct implementation of OCT within commercial spiral-wound and hollow-fiber modules remains challenging due to geometric complexity, there is potential for OCT and related optical techniques to be adapted to diverse membrane configurations and advanced in situ monitoring strategies., supporting its potential scalability for industrial applications [16].

Although OCT has been shown to visualize real-time fouling development on membranes, most previous studies have relied on manual or semi-automated image processing [40,43,47], which may not properly represent fouling heterogeneity or subtle structural changes [16]. Moreover, threshold-based segmentation, which is commonly used in the analysis of OCT images for membrane-fouling studies, suffers from several significant limitations, including sensitivity to image artifacts and variability, arbitrary threshold selection, inability to address complex artifacts, and increased uncertainty with additional processing steps [48,49]. To address these limitations, it is recommended to use post-processing algorithms in combination with automated thresholding methods to improve segmentation consistency and reduce subjectivity [16]. Therefore, developing robust image analysis workflows for OCT data is necessary to enhance reproducibility, extend measurable morphological parameters, and improve the mechanistic understanding of fouling across diverse filtration systems.

The present study seeks to fill a knowledge gap by delivering a unified image-analysis workflow for OCT image data in membrane filtration. Unlike the previous methods based on manual or semi-automated processing, this study proposes an optimized pipeline able to extract quantitative information from OCT data. This approach elevates OCT from a qualitative visualization method to a robust quantitative diagnostic tool, strengthening the mechanistic understanding of fouling dynamics and supporting enhanced control and design of membrane systems. In contrast to previous OCT studies that lacked standardized protocols and thus relied on semi-automated analysis, this study methodically benchmarked various combinations of automated threshold settings. This process successfully established a robust framework for image analysis, applicable across various contamination scenarios.

## 2. Materials and Methods

### 2.1. Optical Coherence Tomography (OCT)

An OCT device (OQ LabScope, Lumedica, Durham, NC, USA) was employed to develop and apply image analysis techniques for microfiltration (MF) fouling. Here, a broadband near-infrared light source centered at approximately 840 nm is split into a sample arm (illuminating the membrane) and a reference arm (fixed-length mirror). Reflected light from the sample and reference arms recombine to produce interference; the interference is dispersed by a spectrometer; a Fourier transform is applied to the measured spectral interferogram to reconstruct a depth profile (A-scan) [36]. Sequential A-scans along a line generate a 2-D cross-section (B-scan); stacking B-scans yield a volumetric (x-y-z) representation of the sample [50].

The OQ LabScope generates 8-bit grayscale images with a matrix size of 512 × 512 pixels. In the acquisition settings used in this study, each pixel represents to a physical size of 3.8 µm in the axial direction, resulting in a nominal imaging depth of approximately 1.95 mm (3.8 µm × 512). This depth aligns with the manufacturer-specified maximum axial scan range of about 2.7–2.8 mm in air (≈2.0 mm in water/tissue). Fouling-layer thickness was calculated from the segmented OCT images using the calibrated axial pixel size (3.8 µm per pixel) and the area-averaging approach described in Section 2.3. The A-scan and B-scan acquisition rates were 34 kHz and 22 B-scans per second, respectively, which are sufficient to capture the temporal evolution of MF fouling under the operating conditions investigated. However, the practical detectability/accuracy for very thin layers is limited by the OCT axial point-spread function, speckle, and interface broadening during segmentation [51,52].

The OCT system used in this study operates in the near-infrared regime, where water is the dominant absorber under the present microfiltration conditions; the relatively low concentrations of humic acid, sodium alginate, and kaolin mainly contribute to scattering and structural contrast rather than strong bulk absorption. The fouling layers were confined to the immediate vicinity of the membrane surface (<20 µm); the local signal-to-noise ratio remained sufficient to delineate the fouling layer despite depth-dependent attenuation. Because the proposed image-processing pipeline relies on relative intensity contrast and automatic thresholding within each B-scan, moderate near-infrared absorption by the aqueous medium was not expected to significantly bias the extracted fouling-layer thickness, although additional calibration may be required for highly absorbing solutes or much higher feed concentrations.

### 2.2. Experimental Setup for Microfiltration (MF) with OCT

**Figure 1:**
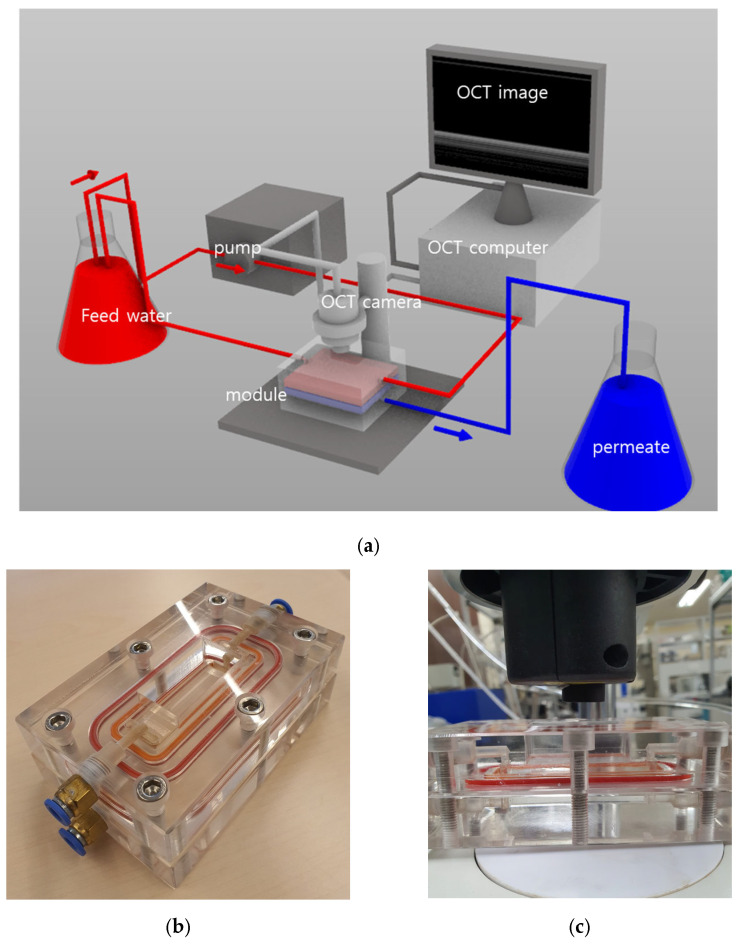
Configuration of the OCT microfiltration setup and the custom membrane cell used for in situ imaging: (**a**) schematic diagram of the MF-OCT system; (**b**) photograph of the transparent membrane cell designed specifically for OCT monitoring; and (**c**) photograph of the same membrane cell assembled under the OCT camera, illustrating the actual imaging setup used during the filtration experiments.

A commercial hydrophilic microfiltration (MF) membrane (GVWP, Merck Millipore, Burlington, MA, USA) with a nominal pore size of 0.22 µm was used in this study. Three feed solutions were used: 50 mg/L humic acid (HA; Sigma-Aldrich, St. Louis, MI, USA); 500 mg/L sodium alginate (alginic acid sodium salt from brown algae; Sigma-Aldrich, USA); and 1000 mg/L kaolin (Sigma-Aldrich, St. Louis, MI, USA). The concentrations of these feed solutions were determined to be optimal based on our preliminary tests. The experimental setup for MF and OCT is illustrated in Figure 1a. Feed water was supplied to the membrane cell using a gear pump (Micropump, Burlington, MA, USA), with the transmembrane pressure maintained at 0.2 bar and the recirculation flow rate in the feed channel held constant at 600 mL/min (corresponding to 0.167 m/s of the crossflow velocity). The feed temperature was kept at 20 ± 1 °C; the permeate flow rate was continuously monitored by a scale (Ohaus, Parsippany, NJ, USA). The membrane cell had a flow channel with the dimensions of 20 mm (W) × 60 mm (L) × 3 mm (H). In each experiment, a new membrane coupon was inserted, giving an effective membrane area of 12 cm^2^.

The photograph in Figure 1b shows the custom-built membrane cell used for OCT imaging. The cell was made of transparent acrylic, providing optical access to the membrane surface during filtration. An OCT probe, which is positioned above the membrane cell (Figure 1c), scanned through the acrylic window and captured real-time images of the membrane surface and developing fouling layer without interrupting filtration. A similar experimental device was used for monitoring RO fouling in our previous work [46].

### 2.3. Image Processing Pipeline

**Figure 2:**
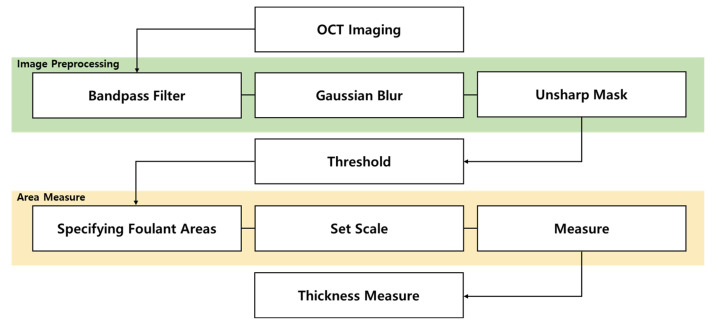
Workflow for OCT-based fouling-thickness extraction.

Figure 2 presents a pipeline for processing OCT images used in this study. At the top, OCT imaging denotes the raw image acquisition from the OCT device. The first stage, which was the image preprocessing, applied a sequence of filtering operations: a band-pass filter, followed by a Gaussian blur, and then an unsharp mask. The purpose of this preprocessing stage was to reduce noise, suppress irrelevant background variations, and enhance edges or structural details within the image. After preprocessing, a threshold operation was applied to distinguish the foulant (deposited material) from the background (membrane or clean areas). This segmentation step converts the processed grayscale image into a binary (or classified) image, where pixels are labeled according to whether they belong to the fouling layer or to the background. Following thresholding, the area and thickness of the fouling layers were measured. A spatial scale was determined based on known pixel-to-physical-size calibration and then by measuring the area occupied by foulants for both measurements. The above image analysis was performed using ImageJ (version 1.53) [53,54]. For each binarized fouling-layer image, the total number of foulant-labeled pixels was quantified and converted to a physical area using the known axial and lateral pixel sizes. The cross-section averaged fouling-layer thickness was subsequently derived by dividing this area by the calibrated horizontal length of the B-scan, so that the resulting value represents the mean thickness over the imaged field of view. The standard deviation from repeated analyses is reported as error bars in the thickness, with time plots to indicate the variability associated with lateral heterogeneity of the cake layer.

## 3. Results and Discussion

### 3.1. MF Fouling by Humic Acid

**Figure 3:**
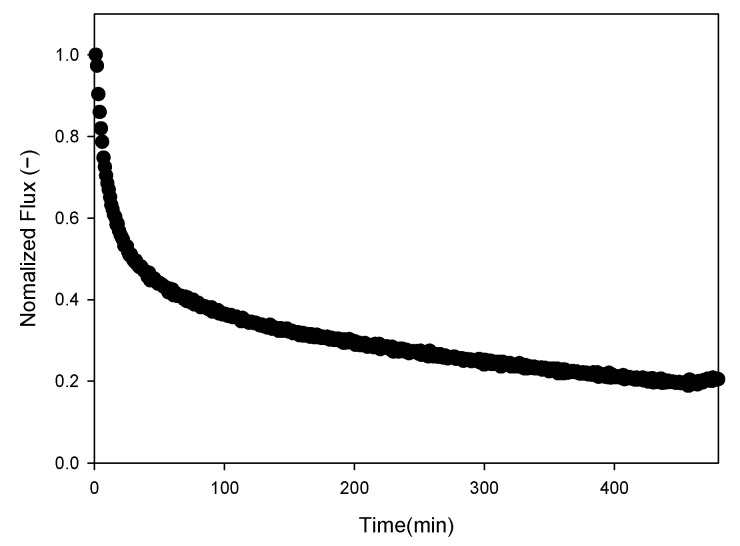
Normalized permeate flux decline during a microfiltration run in the presence of humic-acid fouling (operating conditions: humic acid concentration: 50 mg/L, applied pressure: 0.2 bar).

Figure 3 shows the normalized permeate flux as a function of time during an MF run using humic acid as the model foulant. The flux curve displays a rapid initial decline (31%) within the first 10 min followed by a slower, more gradual decrease over the remaining 400 min, eventually plateauing near 22% of the initial flux. This behavior reflects the classical fouling dynamics: an early stage of rapid fouling-layer formation, transitioning to a slower accumulation that steadily reduces permeate flux as the filtration proceeds.

**Figure 4:**
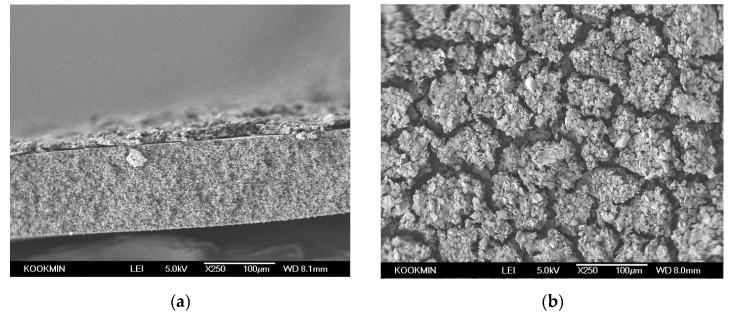
SEM images of a membrane after humic-acid fouling: (**a**) cross-section view reveals the fouling layer deposited on the membrane surface; and (**b**) surface morphology of the fouling layer shows a heterogeneous, granular cake structure formed.

SEM images of the membrane after the MF run are shown in Figure 4. The membrane cross-section is presented in Figure 4a, revealing a distinct surface deposit formed after 8 h of the MF run. Since humic acid forms aggregates in neutral solutions [55], the dominant fouling mechanism evolves to cake formation with filtration time [56]. The top view of the fouling layer is revealed in Figure 4b, highlighting its compact, aggregated morphology. Measurements from the SEM cross-section (Figure 4a) using ImageJ indicate an actual foulant-layer thickness of approximately 15.29 ± 1.54 µm, which serves as the reference for evaluating the accuracy of the OCT-based thickness quantification.

### 3.2. Application of OCT Image Processing Pipeline

**Figure 5:**
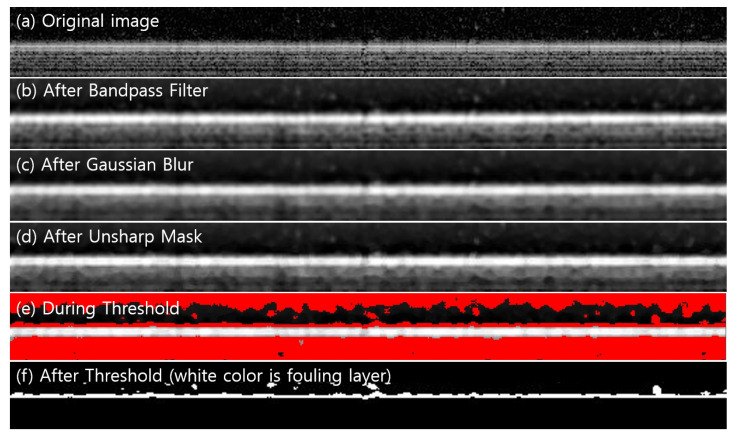
Image processing pipeline for extracting fouling thickness from OCT data: (**a**) the original OCT image; (**b**) after band-pass filtering; (**c**) after Gaussian blur; (**d**) after unsharp-mask sharpening; (**e**) thresholding; and (**f**) the final binary mask.

The image processing pipeline presented in Figure 2 was applied to extract the fouling-layer thickness from the raw OCT data for the MF run. Figure 5a shows the original image as captured, which is in grayscale, with each pixel having a value between 0 and 255. A pixel value of 0 is black, while 255 is white. The values in between are shades of gray that become lighter with larger numbers. The membrane scatters light and is composed of white pixels close to 255. The original OCT image also contained raw back-scatter signals, including noise and background. In Figure 5b, a band-pass filter was used to suppress both low-frequency background variations and high-frequency noise. Applying a bandpass filter can remove noise while preserving the overall appearance of large sections of an image [57]. This feature can also suppress horizontal and vertical streaks caused by scanning the image line by line; therefore, streaks in the membrane cross-section image can be corrected [58].

In Figure 5c, a Gaussian blur (low-pass smoothing) reduced residual noise and smooths the image, improving homogeneity. The Gaussian blur coefficients are determined from the Gaussian filter with a variance of two pixels [59]. In Figure 5d, an unsharp mask was used to enhance edges and boundaries by amplifying high-frequency components. The unsharp mask subtracts a blurred version of the image from the original image to sharpen the edges [59]. This feature reduces noise and sharpens the edges of the blurred image, creating a distinction between the membrane layer, feed water layer, and fouling layer. Finally, in Figure 5e,f, an intensity threshold was applied, first to identify candidate foulant regions (visualized in red), then to produce a binary (black/white) segmentation where white pixels represent the fouling layer. The extracted image was used to determine the area and thickness of the fouling layer.

### 3.3. Effect of Image Preprocessing

**Figure 6:**
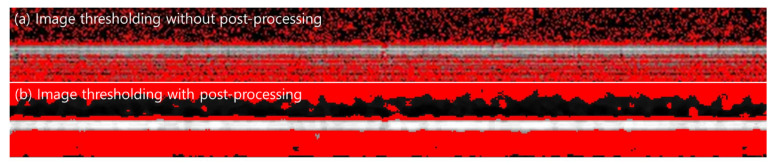
Effect of post-processing on OCT-image threshold segmentation: (**a**) result of direct thresholding; and (**b**) result after applying the full preprocessing pipeline (filtering, smoothing, sharpening) prior to thresholding.

The image-preprocessing step improves the accuracy and reliability of the analysis by reducing noise, which otherwise weakens the performance of thresholding [60]. Figure 6 illustrates this effect by comparing binary OCT segmentation before and after post-processing, showing how noise suppression enables more consistent and physically representative layer detection. Direct automatic thresholding produces substantial noise and fragmented pixels above the membrane surface (Figure 6a), making it difficult to delineate the true cake–membrane interface. The presence of high-intensity spots in the suspension area is misinterpreted as foulants, causing an overestimation of layer thickness. After noise filtering and artifact removal, the cake layer boundary becomes continuous and geometrically representative of the actual fouling-layer structure (Figure 6b). This improvement enables more reliable quantification of cake thickness and reduces algorithmic bias introduced by noisy images.

### 3.4. Thresholds for Extracting Fouling Layers

Thresholding is a fundamental technique for separating objects from the background by mapping pixel intensities onto a 0–255 histogram and keeping only values within a selected range [61]. Although users can adjust thresholds visually, manual tuning is inefficient and highly operator-dependent, especially for complex image sets [62]. Automatic thresholding provides a more consistent alternative, but selecting an appropriate algorithm is essential because OCT membrane cross-sections contain heterogeneous speckle and low-contrast interfaces [63,64]. To determine a robust threshold for fouling-layer extraction, this study applied all 17 automatic global thresholds available in ImageJ: default, Huang [65], Intermodes [66], IsoData [67], IJ_IsoData, Li [68], MaxEntropy [69], Mean, MinError [70], Minimum [66], Moments [71], Otsu [72], Percentile [63], RenyiEntropy [64], Shanbhag [73], Triangle [74] and Yen [75], which are provided by ImageJ.

**Figure 7:**
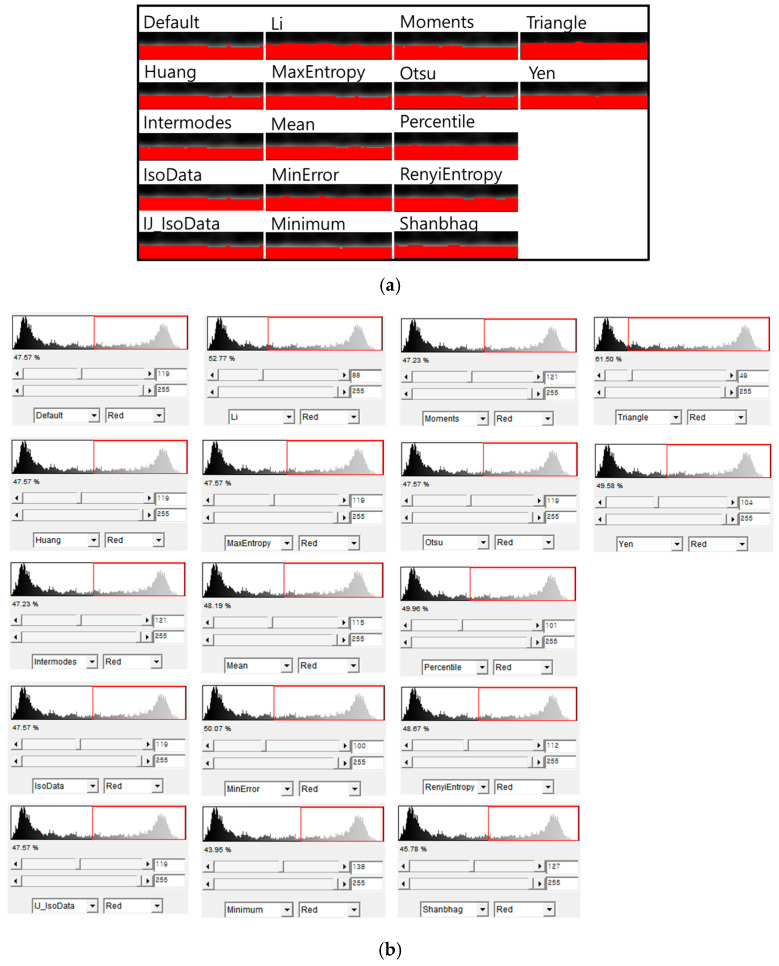
Comparison of different automatic thresholding methods applied to OCT images: (**a**) segmented images by different methods (in red); and (**b**) corresponding intensity histograms.

The segmentation results differ markedly across the 17 automatic thresholding algorithms because each method interprets the OCT intensity histogram according to its own statistical or entropy-based criteria. As shown in Figure 7, some algorithms (e.g., Triangle, Yen) select relatively high threshold values, producing thinner segmented layers, while others (e.g., Minimum, Intermodes) choose much lower thresholds and therefore classify a larger portion of the image as fouling. Methods such as Otsu, MaxEntropy, and RenyiEntropy tend to emphasize the separation between dominant intensity peaks but are sensitive to the broad speckle distribution characteristic of OCT data. In contrast, mean-based or percentile-based thresholds generate a more conservative segmentation but may underrepresent low-contrast fouling regions. This variability demonstrates that no single thresholding algorithm performs uniformly across OCT images, reinforcing the need for algorithm selection tailored to the image characteristics and analysis objective.

**Figure 8:**
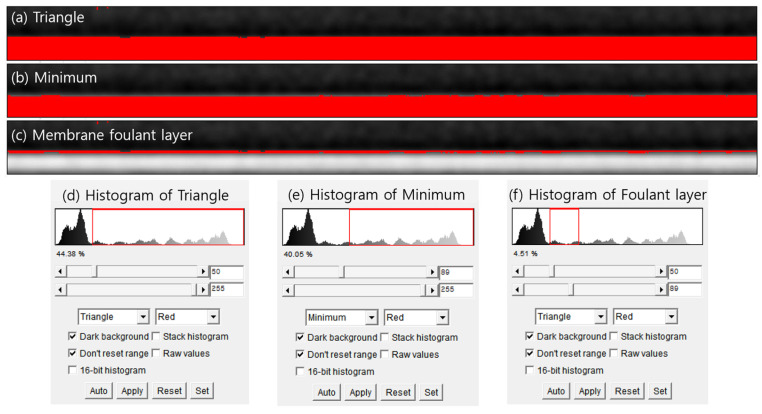
OCT images and corresponding histograms under different automatic thresholding schemes: (**a**) segmented fouling layer (in red) using the Triangle method; (**b**) segmented layer using the Minimum method; (**c**) resulting isolated fouling region by the two methods. (**d**) histogram of triangle; (**e**) histogram of minimum; and (**f**) histogram of foulant layer.

In order to extract the images of the fouling layer, it is necessary to establish two automatic thresholds. The first threshold was applied to separate the fouling layer from the feedwater region; the other was used to separate the fouling layer from the membrane surface. Each automatic threshold is computed algorithmically and defines the range of pixel intensities included in a segmented region. For example, Figure 8 illustrates the procedure used to isolate the fouling layer by combining two automatically generated thresholds, which are the triangle and minimum thresholding methods, respectively. As illustrated in Figure 8a, the first threshold (Triangle) is applied, capturing the interface between the feedwater and the fouling layer. The corresponding histogram is presented in Figure 8d. Figure 8b illustrates the second threshold (Minimum) employed to differentiate the membrane from the overlying fouling layer; its histogram is presented in Figure 8e. The membrane-foulant threshold should be subtracted from the feed-foulant threshold to eliminate all but the intensity range corresponding to the fouling layer. The resulting isolated fouling region is shown in Figure 8c, with its associated histogram in Figure 8f. In this study, combinations of the 17 thresholding algorithms were considered in exploring the optimum one for extracting the fouling layer from the OCT image.

**Figure 9:**
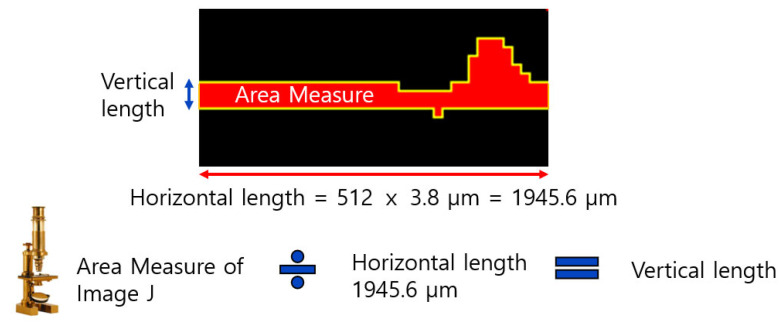
Illustration of the procedure for deriving average fouling-layer thickness from OCT segmentation.

After extracting the fouling layer, the segmented fouling region was converted into an average fouling-layer thickness using the following method, as illustrated in Figure 9. First, the cross-sectional area of the fouling layer was computed in units of pixels. The horizontal dimension of the OCT image is fixed at 512 pixels, corresponding to 1945.6 µm based on the system’s lateral resolution (3.8 µm per pixel). Dividing the measured area by this calibrated horizontal length yields the mean vertical thickness of the fouling layer. This approach transforms a two-dimensional area measurement into an average one-dimensional thickness, enabling consistent quantification across images.

### 3.5. Selection of Optimum Automatic Thresholding Algorithms

Table 1 presents the fouling-layer areas obtained after applying each of the 17 automatic thresholding algorithms to three independently acquired OCT images taken at the 8 h operating point. Because OCT suffers from mechanical variation and speckle-induced noise, images captured at the same time exhibit slight differences in their intensity histograms. Consequently, each automatic algorithm computes a different threshold for each image, leading to variability in the extracted fouling area. By preprocessing all images identically and then applying the same thresholding functions, the table quantifies how sensitive each algorithm is to these small image-to-image fluctuations. The spread in area measurements across the three replicates illustrates the inherent variability of automatic thresholding and highlights its influence on the reliability of fouling-thickness estimation.

**Figure 10:**
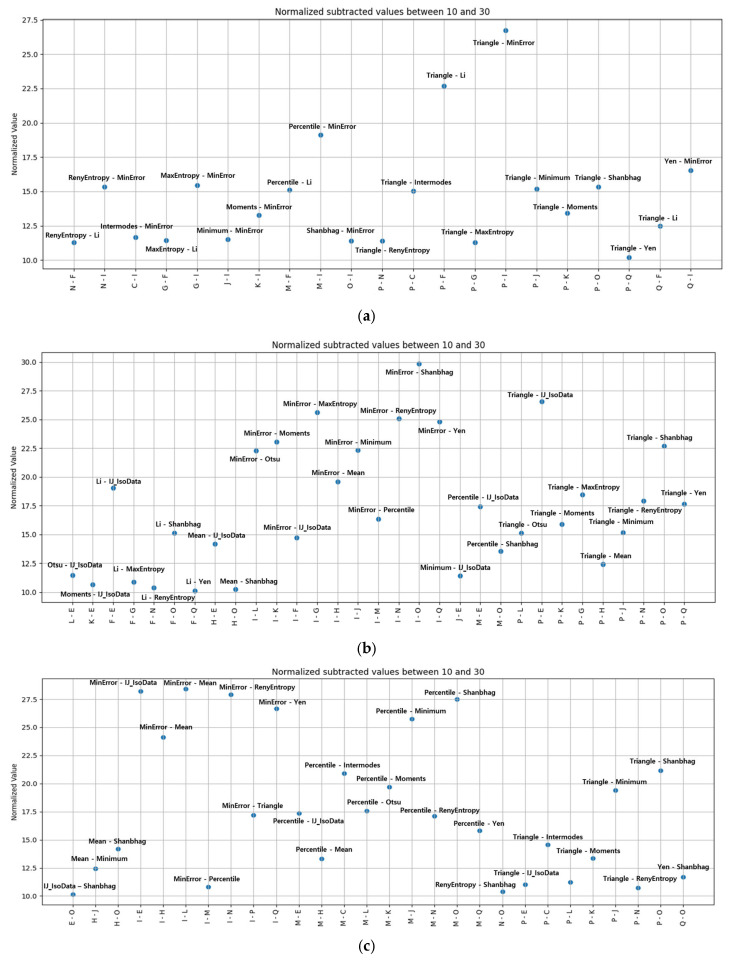
Fouling-layer thickness obtained using different auto-threshold methods: (**a**) Case 1; (**b**) Case 2; and (**c**) Case 3.

Figure 10 presents the fouling-thickness values obtained by pairing automatic thresholds and assessing their performance across three OCT images. From the 17 available algorithms, all 289 possible threshold pairs were evaluated; each pair was applied sequentially in the fouling-layer subtraction calculation. Only results yielding a normalized thickness between 10 and 30 µm, which is the range expected from the SEM reference measurement (15.29 ± 1.54 µm), were plotted. Figure 10a–c correspond to the three OCT images, with each point representing a unique threshold pair. The distributions show that most combinations either fall outside the acceptable range or exhibit substantial variation between images, indicating limited robustness. Only four threshold pairs, including Triangle–Shanbhag, Triangle–Minimum, Triangle–Moments, and Triangle–RenyiEntropy, consistently produced values within the 10–30 µm range for all three images. Their mean thicknesses and standard deviations are reported in Table 2. Among these, Triangle–Shanbhag yielded the largest thickness estimate (19.72 ± 3.17 µm), while Triangle–RenyiEntropy produced the smallest (13.36 ± 3.24 µm). The relatively low standard deviations demonstrate that these threshold pairs provide reproducible measurements despite image-to-image variability.

**Figure 11:**
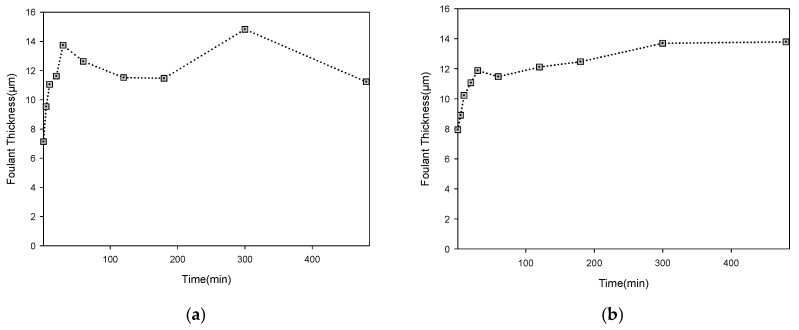

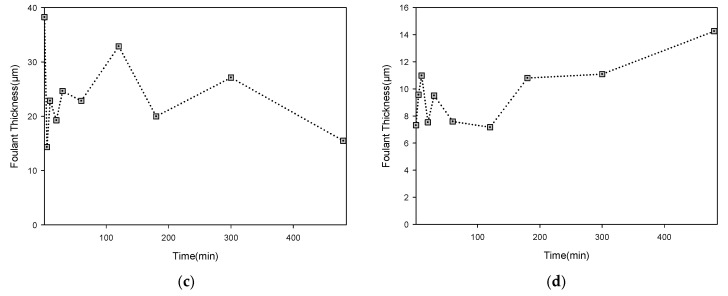
Evolution of fouling-layer thickness during MF operation under different automatic-threshold schemes: (**a**) thickness profile using the Triangle–Minimum threshold combination; (**b**) using Triangle–Moments; (**c**) using Triangle–Shanbhag; and (**d**) using Triangle–RenyiEntropy.

Figure 11 compares the temporal evolution of fouling-layer thickness during the 8 h humic-acid MF operation (Figure 3) using the four threshold pairs that exhibited stable error levels against SEM measurements. The OCT analyses produced a consistent baseline thickness of 7–12 µm at t = 0 across all four methods. This value corresponds to only 2–3 pixels in the reconstructed images, which indicates that the apparent thickness arises from the OCT system’s axial resolution limit and the interface-reconstruction procedure rather than from immediate cake formation [16,76]. During filtration, fouling develops progressively through adsorption and concentration polarization; values near the resolution limit are strongly affected by image noise and the behavior of automatic threshold algorithms. These constraints make it impractical to identify a single, definitive time point at which the fouling layer first appears in the OCT image. This study therefore reports the thickness—time curves without artificially resetting the initial thickness to zero or designating an assumed onset of cake formation [77]. The curves were subsequently interpreted alongside the corresponding flux decline pattern to examine how fouling-layer growth and filtration performance evolve over time.

All thickness profiles were evaluated alongside the observed flux decline, which showed a 79.5% reduction over 8 h and a rapid 57.5% drop within the first hour (Figure 3). Among the four threshold combinations, only Triangle–Moments (Figure 11b) produced a monotonic increase in fouling thickness that parallels the continuous flux decline. In contrast, Triangle–Minimum, Triangle–Shanbhag, and Triangle–RenyiEntropy (Figure 11a,c,d) yielded fluctuating thickness values with no consistent trend, indicating reduced suitability for time-dependent fouling analysis. The Triangle–Moments curve shows a steady accumulation of the fouling layer, with a 73.6% increase in thickness by the end of the 8 h run. The increase during the first hour was 44.5%, mirroring the sharp early-stage flux decline. This alignment between fouling growth and flux loss indicates that rapid cake-layer development in the initial phase of MF operation is the dominant mechanism driving the early flux deterioration.

Triangle is a geometric binarization method that identifies a threshold by exploiting histogram skewness [60,74]. The algorithm assumes that pixel intensities cluster near one end of the histogram and projects a line from the histogram’s maximum peak toward the opposite tail; the threshold is then selected at the point of greatest deviation from this line [57]. In contrast, the Moments method determines the threshold by preserving statistical moments of the histogram distribution [69]. This approach has been shown to outperform many other automatic thresholds in diverse imaging applications. For example, the Moments method has been reported to be one of the most effective algorithms for OCT image analysis in macular research [78] and cell counting [56]. These findings, together with the present results, indicate that automatic thresholding methods—particularly Triangle and Moments—are well suited for segmenting OCT images of the fouling layer.

### 3.6. Real-Time Inorganic Fouling Analysis

**Figure 12:**
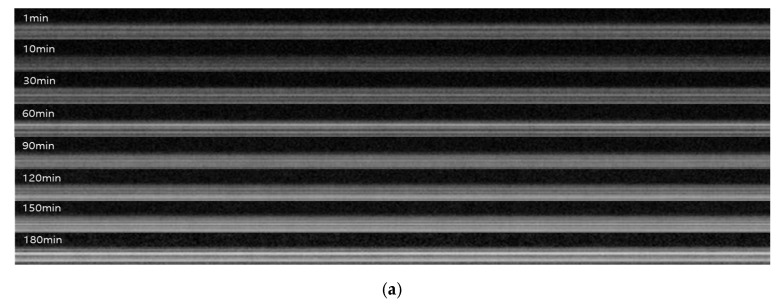

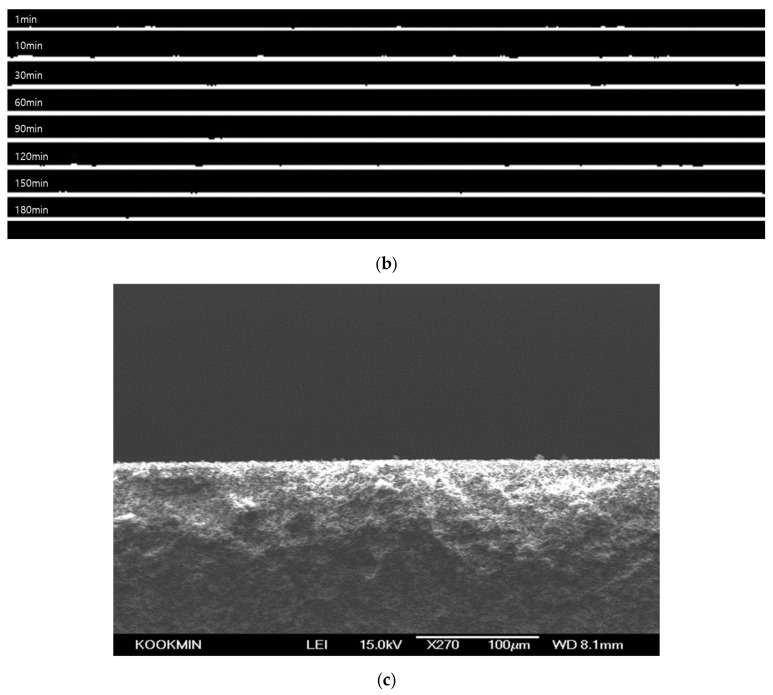
Time series of OCT images and corresponding fouling-layer segmentations during membrane operation of kaolin: (**a**) original OCT cross-section images acquired at various filtration times; (**b**) corresponding binary images after thresholding, with the fouling layer shown in white; and (**c**) SEM image of kaolin fouling after operation.

Figure 12 illustrates the application of the optimized foulant-extraction method to an inorganic fouling experiment using kaolin. A high kaolin concentration (1000 ppm) was selected to induce rapid cake formation, as kaolin particles readily accumulate on membrane surfaces and generate compact, dense deposits [79]. Figure 12a shows the raw OCT cross-sectional images acquired over 3 h of MF operation. As filtration proceeds, increasing grayscale intensity appears near the membrane surface and within the feedwater region, reflecting the presence and accumulation of suspended kaolin particles. The corresponding foulant-layer images obtained after image preprocessing and automatic thresholding are shown in Figure 12b. The white regions represent the extracted cake layer, enabling clear visualization of fouling progression over time.

**Figure 13:**
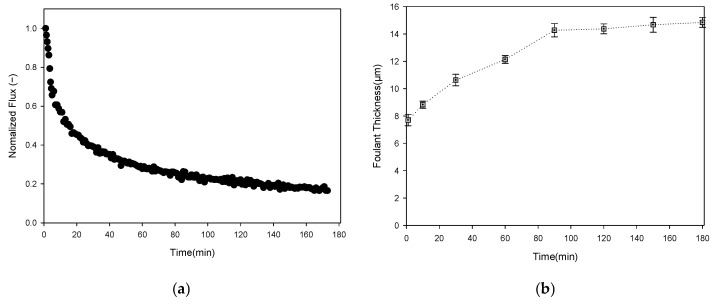
Comparison of flux decline and fouling-layer growth during MF Kaolin operation: (**a**) normalized permeate flux as a function of filtration time; and (**b**) kaolin fouling-layer thickness (µm) determined from OCT images.

The flux decline during the 3 h MF operation with the kaolin feed was compared with the evolution of fouling-layer thickness. A rapid drop in normalized flux was observed in Figure 13a. The flux decreased by approximately 43% within the first 10 min (from 634.3 to 361.7 L/m^2^·h). By the end of the run, the flux reached 86.6 L/m^2^·hr, corresponding to an 86.4% reduction relative to the initial value.

As shown in Figure 13b, the corresponding fouling-layer thickness was derived from the threshold-based OCT analysis. To account for lateral heterogeneity, each B-scan was subdivided into three equal-width regions of interest (ROIs) along the horizontal direction; the average fouling-layer thickness was calculated for each ROI separately. The standard deviations of the three measurements were then reported as error bars in the thickness–time plots to reflect the variability introduced by the lateral non-uniformity of the cake layer. The fouling layer increased from 7.7 µm at 1 min to 11.3 µm at 10 min, representing a 48% rise during the same period in which the steepest flux decline occurred. After 30 min, the fouling thickness stabilized at approximately 11.5 ~ 11.6 µm and remained nearly constant for the remainder of the experiment. The combined results indicate that the majority of cake layer formation occurred within the first 10 min of operation; this early rapid deposition was the primary driver of the sharp initial flux loss. Subsequent flux decline was minimal, consistent with the near-steady fouling thickness observed after 30 min. These results demonstrate that the proposed image processing approach effectively tracks the progression of cake formation and provides valuable insight into the dominant fouling mechanisms.

In the kaolin fouling experiment, SEM cross-section analysis was performed to qualitatively support the OCT-derived thickness results (Figure 12c). However, because the inorganic kaolin cake is inherently mechanically fragile, partial detachment of the fouling layer can occur during membrane disassembly and cross-sectional sample preparation. As a result, the SEM image may visually under-represent the original fouling structure formed during filtration, which limits the reliability of absolute thickness determination from SEM alone. Based on the regions that remained attached, the measured cake thickness was 12.2 ± 3.5 µm. The corresponding OCT-derived mean thickness was 14.8 ± 0.36 µm, which falls within the SEM uncertainty despite the partial detachment. This outcome highlights a practical limitation of SEM for fragile cake layers—destructive preparation can disturb the deposit and bias thickness estimates—whereas OCT enables non-destructive, real-time tracking of fouling-layer growth without sample disturbance, providing more robust thickness quantification under operating conditions.

### 3.7. Real-Time Gel Fouling Analysis

**Figure 14:**
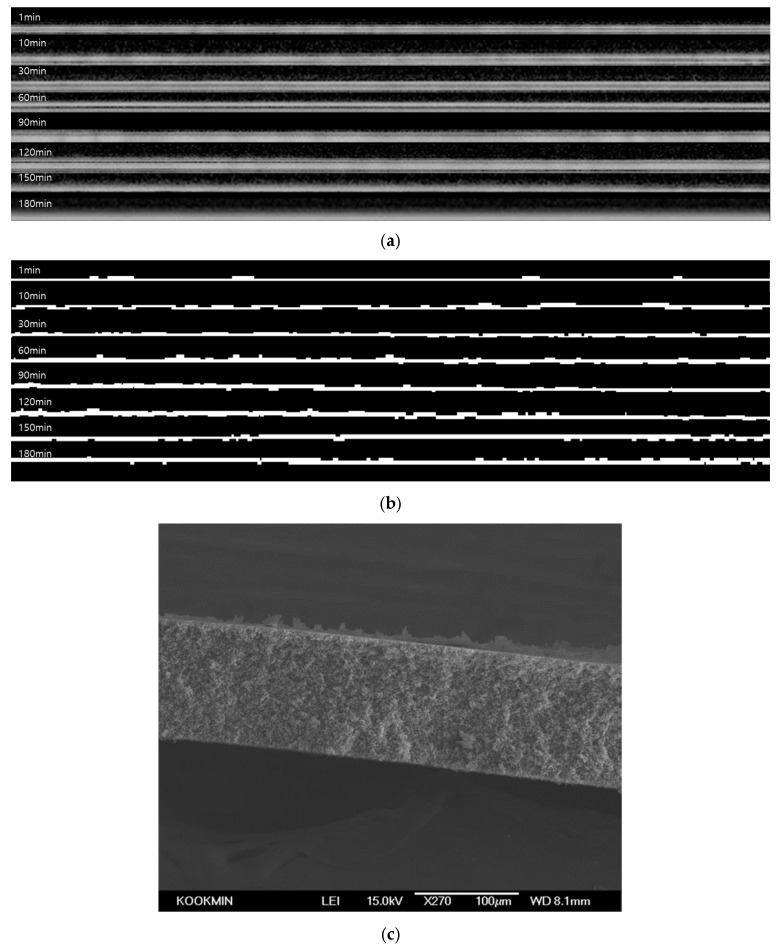
Time-series of OCT images and corresponding fouling-layer segmentations during membrane operation of sodium alginate: (**a**) original OCT cross-section images acquired at various filtration times; (**b**) corresponding binary images after thresholding, with the fouling layer shown in white; and (**c**) SEM image of sodium alginate fouling after operation.

Figure 14 illustrates the application of the optimized foulant-extraction method to an organic gel-type fouling experiment using sodium alginate. A feed concentration of 500 ppm sodium alginate was selected to induce the formation of a hydrated polysaccharide gel layer, which is widely used as a model biopolymer foulant in membrane operation. Figure 14a shows the raw OCT cross-sectional images acquired over 3 h of MF operation. As filtration proceeds, a progressively brighter and more continuous high-intensity band develops near the membrane surface, whereas the intensity changes in the bulk feedwater region remain relatively moderate. This behavior indicates the progressive formation and consolidation of a thin, dense gel layer rather than a granular particulate cake. The corresponding foulant-layer images obtained after image preprocessing and automatic thresholding are presented in Figure 14b. The white regions represent the extracted cake layer, enabling clear visualization of fouling progression over time. Figure 14c, an SEM image, is presented with the same scale bar as that shown in Figure 12c. Nevertheless, minor differences in the apparent magnification may arise from variations in sample positioning relative to the detector during SEM mounting. Such effects can influence the visual appearance of fouling layers without altering the actual length calibration.

**Figure 15:**
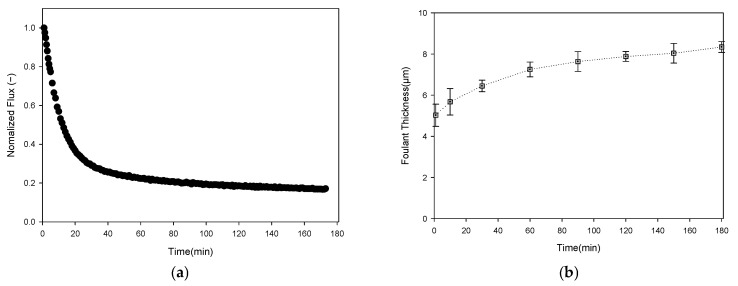
Comparison of flux decline and fouling-layer growth during MF sodium alginate operation: (**a**) normalized permeate flux as a function of filtration time; and (**b**) sodium alginate fouling-layer thickness (µm) determined from OCT images.

The flux decline during the 3 h MF operation with the sodium alginate feed was compared with the evolution of fouling-layer thickness. As shown in Figure 15a, the normalized flux exhibited a sharp initial decrease followed by a much more gradual decline. The flux dropped by approximately 43% within the first 10 min (from 355.3 to 202.3 L/m^2^·h). By the end of the run, the flux reached 59.6 L/m^2^·h, corresponding to an overall reduction of about 83% relative to the initial value. After the MF operation, cross-sectional SEM images were obtained to qualitatively examine the structure of the fouling layer. The SEM micrographs confirmed the presence of a thin, compact and relatively smooth gel layer attached to the membrane surface, which is clearly distinct from the granular kaolin cake. As shown in Figure 14c, for the sodium alginate (SA) fouling experiment, the white scale bar corresponds to 100 µm. Based on this calibration, multiple local thickness measurements were performed using ImageJ. Consequently, the SEM-based average fouling-layer thickness was determined to be 11.3 ± 4.2 µm. Although there is a slight difference between the SEM and OCT measurements. this can be attributed to the average value difference caused by the heterogeneous nature of the contamination layer. The SEM results show a large standard deviation, while the OCT results exhibit a low standard deviation value. This proximity validates the reliability of the OCT method for analyzing gel-type fouling.

As shown in Figure 15b, the corresponding fouling-layer thickness was derived from the threshold-based OCT analysis using the same image-processing workflow and the optimized Triangle–Moments pair. The gel-layer thickness increased from 5.0 (±0.54) µm at 1.5 min to 5.68 (±0.64) µm at 10 min, and then to 7.25 (±0.36) µm at 60 min, representing a net increase of 2.23 µm (approximately 44% relative to the initial value). During this period, the highest growth rate occurred between 1.5 and 10 min with the steepest flux decline. After 60 min, the increase in gel thickness became much slower; the layer gradually approached a value of 8.3 (±0.26) µm at 180 min. On average, 67% of the total thickness growth occurred within the first 60 min. Only a modest additional increase (0.70 µm) was observed between 90 and 180 min, which may be attributed to local compaction or restructuring of the gel matrix. The combined results indicate that most of the sodium alginate gel layer formed during the early stage of operation and that this rapid initial gel deposition was the primary driver of the sharp early flux loss. Subsequently, flux deterioration was relatively modest, consistent with the nearly steady fouling thickness observed after about 90 min.

## 4. Conclusions

This study demonstrates that OCT, combined with an optimized image processing workflow, enables quantitative, real-time characterization of membrane cake fouling. By applying a standardized preprocessing sequence and evaluating all 17 automatic thresholding algorithms available in ImageJ, the Triangle–Moments combination was identified as the most reliable segmentation method. Among the 289 possible threshold pairs tested, only four produced thickness values within the physically validated range of 10–30 µm across all three OCT images; Triangle–Moments showed the lowest variability (14.23 ± 1.18 µm). Validation using SEM confirmed that OCT-derived thickness estimates closely matched the measured humic-acid cake thickness of 15.29 (±1.54) µm.

Application of the method to humic acid fouling (8 h) quantified a 73.6% increase in cake thickness and captured a 44.5% rise within the first hour, mirroring the 57.5% early flux drop and the overall 79.5% flux decline after 8 h. In kaolin fouling (3 h), the approach detected a rapid early increase from 7.7 µm to 11.3 µm (a 48% rise in 10 min), followed by stabilization at ~11.5 µm, while flux continued to deteriorate to 86.6 L m^−2^ h^−1^ (an 86.4% decline). In sodium alginate gel fouling (3 h), the same workflow tracked a thin, dense gel layer that increased from 5.0 ± 0.54 µm to 8.3 ± 0.26 µm, with ~67% of the total thickness increase occurring within the first 60 min, consistent with the pronounced early flux loss (~43% within 10 min) and the overall ~83% decline by the end of the run. Together, these case studies demonstrate that the method quantifies the magnitude and rate of foulant layer growth. It also helps identify periods when flux decline is growth-dominated versus periods when flux continues to decrease despite minimal additional thickness increase.

Overall, the proposed workflow, including preprocessing and dual-threshold subtraction, provides a quantitative diagnostic tool for tracking fouling development, interpreting flux behavior, and supporting operational decision-making. These image processing principles can be extended to diagnose contamination in UF, RO, and MD systems when OCT imaging of membrane surfaces is possible. Although this proposed workflow is general, quantitative performance can be affected by membrane optical scattering/reflectivity and foulant type/concentration. Therefore, when applying the method to a new membrane or feed, it is necessary to re-confirm pixel-to-length calibration, verify that preprocessing parameters preserve the membrane–cake interface, and re-screen threshold-pair performance using a small representative image set. If possible, an external thickness reference, such as SEM, may be used for at least one endpoint.

Further work is required to optimize the OCT-based method for different membrane configurations, including hollow-fiber and spiral-wound modules. Additionally, this framework must be extended to 3D imaging to resolve internal fouling and spatial heterogeneity within the cake layer. A systematic charge-class validation, including a cationic and a neutral model foulant under controlled pH/ionic strength, is also needed in future work.

## Figures and Tables

**Table 1:** Automatic-threshold segmentation results for fouling-area measurements.

Sample Name	Auto Threshold	Area Measure 1 (µm^2^)	Area Measure 2 (µm^2^)	Area Measure 3 (µm^2^)
A	Default	51,752.96	66,539.52	43,854.28
B	Huang	29,457.6	66,539.52	51,781.84
C	Intermodes	50,222.32	59,435.04	36,966.4
D	IsoData	51,752.96	66,539.52	43,464.4
E	IJ_IsoData	29,457.6	66,539.52	43,854.28
F	Li	66,467.32	44,561.84	51,781.84
G	MaxEntropy	45,254.96	66,785	39,334.56
H	Mean	56,994.68	66,539.52	51,781.84
I	MinError	95,072.96	36,735.36	98,740.72
J	Minimum	51,651.88	59,146.24	27,573.12
K	Moments	50,222.32	62,597.4	39,334.56
L	Otsu	51,752.96	66,539.52	43,464.4
M	Percentile	63,333.84	73,932.8	77,672.76
N	RenyEntropy	46,280.2	66,539.52	44,403
O	Shanbhag	37,024.16	58,900.76	24,143.68
P	Triangle	81,152.8	88,719.36	65,312.12
Q	Yen	46,814.48	68,878.8	46,857.8

**Table 2:** Fouling-layer thickness derived via automatic-threshold segmentation.

Sample Name	Auto Threshold	Thickness
P-O	Triangle–Shanbhag	19.72 (±3.17)
P-J	Triangle–Minimum	16.59 (±1.99)
P-K	Triangle–Moments	14.23 (±1.18)
P-N	Triangle–RenyEntropy	13.36 (±3.24)

## Data Availability

The original contributions presented in this study are included in the article. Further inquiries can be directed to the corresponding author.

## Abbreviations

The following abbreviations are used in this manuscript:

AFM	Atomic Force Microscopy
A-scan	Axial scan (depth-resolved OCT signal)
B-scan	Cross-sectional OCT image composed of sequential A-scans
CC BY	Creative Commons Attribution
DOTM	Direct Observation Through the Membrane
EIS	Electrical Impedance Spectroscopy
HA	Humic Acid
ImageJ	Image Processing and Analysis in Java
MD	Membrane Distillation
MF	Microfiltration
MOF	Metal–Organic Framework
NIR	Near-Infrared
NMR	Nuclear Magnetic Resonance
OCT	Optical Coherence Tomography
PSF	Point-Spread Function
ROI	Region of Interest
RO	Reverse Osmosis
SA	Sodium Alginate
SEM	Scanning Electron Microscopy
UF	Ultrafiltration
UTDR	Ultrasonic Time-Domain ReflectometryMultidisciplinary Digital Publishing Institute
